# Positive impact of the participation in the ENCHANTED trial in reducing Door-to-Needle Time

**DOI:** 10.1038/s41598-017-14164-8

**Published:** 2017-10-26

**Authors:** Jie Yang, Xia Wang, Jian ping Yu, Jing Hang, Pablo Lavados, Thompson Robinson, Hisatomi Arima, Richard I. Lindley, Craig S. Anderson, John Chalmers

**Affiliations:** 1 Department of Neurology, the First Affiliated Hospital of Chengdu Medical College, Chengdu, China; 2 The George Institute for Global Health, Faculty of Medicine, University of New South Wales, Sydney, Australia; 3 Department of Neurology, Nanjing First Hospital, Nanjing Medical University, Nanjing, China; 4 Servicio de Neurología, Departamento de Medicina, ClínicaAlemana, Universidad del Desarrollo, and Universidad de Chile, Santiago, Chile; 5 Department of Cardiovascular Sciences and NIHR Biomedical Research Centre, University of Leicester, Leicester, UK; 6 Department of Public Health, Fukuoka University, Fukuoka, Japan; 7 The George Institute for Global Health at Peking University Health Science Center, Beijing, China

## Abstract

Door-to-needle time (DNT) is a key performance indicator for efficient use of intravenous thrombolysis in acute ischemic stroke (AIS). We aimed to determine whether DNT improved over time in the Enhanced Control of Hypertension and Acute Stroke Study (ENCHANTED) and the clinical predictors of DNT. Temporal trends in DNT were assessed across fourths of time since activation of study centers using generalized linear model. Predictors of long DNT (>60 min) were determined in logistic regression models. Overall mean DNT (min) was 71.8 (95% confidence interval [CI] 70.4–73.2), but decreased significantly over time (fourths): 77.9 (74.9–80.9), 69.3 (66.7–72.0), 69.1 (66.5–71.8) and 71.4 (68.7–74.2) (P for trend, 0.003). The reduction in DNT was particularly marked in China (P for trend, 0.001), but was not significant across the other participating countries (P for trend, 0.065). Independent predictors of long DNT were recruitment from China, short onset-to-door time, lower numbers of patients treated per center, higher diastolic blood pressure, off-hour admission, and absence of proximal clot occlusion. DNT in ENCHANTED declined progressively during the trial, especially in China. However, DNT in China is still longer than the key performance parameter of ≤60 minutes recommended in guidelines. Effective national programs are needed to improve DNT in China.

## Introduction

Intravenous recombinant tissue plasminogen activator (rtPA) is the only approved medical reperfusion therapy for patients with acute ischemic stroke (AIS)^1^. Guidelines^1,2^ recommend this treatment to be initiated as soon as possible within 4.5 hours of stroke onset, as shorter onset-to-treatment time (OTT) translates into a better functional outcome^3,4^. OTT is composed of onset-to-door time (ODT) and door-to-needle time (DNT). However, it is difficult to accomplish improvement of ODT because campaigns aiming at raising public awareness of stroke symptoms have only limited impact on ODT^5^. Conversely, a focus on decreasing in-hospital DNT is feasible, valuable and arguably essential, for quality systems of stroke care^1,6^.

We used the dataset from the Enhanced Control of Hypertension and Acute Stroke Study (ENCHANTED)^7,8^ rtPA-dose comparison arm to determine (i) whether DNT improved over time among participants, (ii) the extent to which DNT in China differed from other regions, and (iii) the clinical predictors of DNT.

## Results

There were 3219 (97.3%) patients with available data were included in these analyses. Table 1 shows the baseline characteristics of patients by DNT (≤60 vs. >60 mins). The majority of participants (1903 [59.1%]) had DNT >60 m. These were significantly more likely to be younger, Chinese, hypertensive, off-hour admission, being treated in a larger center, and have longer ODT. Multivariable analysis shows the independent predictors of longer DNT included recruitment from China (OR 9.75, 95%CI 7.70–12.35; P < 0.0001), shorter ODT (0.58, 0.53–0.65; P < 0.0001), fewer patients treated in the center (0.96, 0.95–0.97; P < 0.0001), higher diastolic blood pressure (BP) (1.07, 1.00–1.14; P = 0.037), off-hour admission (1.27, 1.07–1.50; P = 0.005), and CT or MRI angiogram not showing proximal occlusion (0.65, 0.52–0.82; P = 0.0002). Chinese patients were twice as likely (1147, 83.0%) to have DNT >60 m compared to non-Chinese patients (756, 41.2%). Shorter ODT and fewer patients treated in the center were independently associated with DNT >60 m for both Chinese and non-Chinese patients. Among non-Chinese patients, male patients (female vs. male: 0.74 [0.61–0.90]; P = 0.003) and Asians (other than Chinese) (0.62, 0.51–0.76; P < 0.0001) were less likely to have DNT >60 m (see Supplementary Tables S1 and S2).

**Table 1:** Baseline characteristics of patients with acute ischemic stroke, according to door-to-needle time above and below 60 minutes for use of intravenous thrombolysis treatment.

	DNT ≤60 min	DNT >60 min	*P* value	OR (95%CI)	aOR (95%CI)	*P* value
	N = 1316	N = 1903				
Age, years	68 (13)	66 (13)	<0.0001	0.89 (0.85–0.95)*		
Female	479 (36)	742 (39)	0.136			
Region of recruitment			<0.0001			
China	235 (18)	1147 (60)		6.98 (5.90–8.26)	9.75 (7.70–12.35)	<0.0001
Others	1081 (82)	756 (40)		1.0		
Clinical features						
Systolic BP, mmHg	149 (20)	150 (19)	0.059			
Diastolic BP, mmHg	83 (13)	86 (13)	<0.0001	1.18 (1.12–1.25)*	1.07 (1.00–1.14)*	0.037
Heart rate, beats per minute	80 (16)	79 (15)	0.156			
NIHSS score	8 (5–13)	8 (5–14)	0.523			
≥14	315 (24)	516 (27)	0.043	1.18 (1.01–1.39)		
GCS score	15 (14–15)	15 (13–15)	0.0003			
Severe (3–8)	33 (3)	99 (5)	0.0002	2.13 (1.43–3.18)		
Medical history						
Hypertension	822 (63)	1194 (63)	0.871			
Previous stroke	177 (13)	292 (15)	0.134			
Coronary artery disease	103 (8)	130 (7)	0.284			
Other heart disease	191 (15)	270 (14)	0.796			
Atrial fibrillation	271 (21)	353 (19)	0.151			
Diabetes mellitus	261 (20)	368 (19)	0.728			
Hypercholesterolemia	291 (22)	255 (13)	<0.0001	0.55 (0.45–0.66)		
Current smoker	311 (24)	447 (24)	0.919			
Pre-stroke function on the mRS						
No symptoms	1059 (81)	1561 (82)	0.238			
No significant disability	257 (20)	340 (18)				
Medications						
Antihypertensive agents	635 (48)	826 (43)	0.007	0.82 (0.71–0.95)		
Warfarin anticoagulation	32 (2)	46 (2)	0.971			
Aspirin/other antiplatelet agent(s)	344 (26)	388 (24)	0.0001	0.72 (0.61–0.85)		
Glucose-lowering treatment	164 (13)	238 (13)	0.989			
Statin/other lipid lowering	337 (26)	267 (14)	<0.0001	0.47 (0.40–0.57)		
Brain imaging features	N = 1316	N = 1903				
Visible early ischemic changes	369 (28)	390 (21)	<0.0001	0.66 (0.56–0.78)		
Visible cerebral infarction	341 (26)	387 (20)	0.0002	0.73 (0.62–0.86)		
Visible cerebral infarction with mass effect	19 (1)	28 (2)	0.949			
CT/MRI angiogram shows proximal occlusion	295/1290 (23)	203/1888 (11)	<0.0001	0.41 (0.33–0.49)	0.65 (0.52–0.82)	0.0002
Final diagnosis at time of hospital separation	N = 1300	N = 1877				
Non-stroke	51 (4)	34 (2)	<0.0001			
Large artery occlusion due to significant atheroma	421 (32)	833 (44)		1.0		
Small vessel or perforating vessel lacunar disease	261 (20)	404 (22)		0.78 (0.64–0.95)		
Cardioembolism	301 (23)	331 (18)		0.56 (0.46–0.68)		
Dissection	15 (1)	10 (1)		0.52 (0.43–0.64)		
Other or uncertain etiology	251 (19)	265 (14)				
Off-hour admission†	627 (48)	1153 (61)	<0.0001	1.69 (1.47–1.95)	1.27 (1.07–1.50)	0.005
No. of patients treated in the center	57 (33–106)	96 (27–156)	<0.0001	1.04 (1.03–1.05)	0.96 (0.95–0.97)	<0.0001
Time from onset to door, hour	1.5 (1.0–2.3)	1.2 (0.8–1.9)	<0.0001	0.63 (0.58–0.68)	0.58 (0.53–0.65)	<0.0001

Data are n (%), mean (SD), or median (IQR)· P values based on Chi-square, T test, or Wilcoxon signed-rank test.

aOR denotes adjusted odds ratio, BP blood pressure, CI confidence interval, CT computerized tomography, GCS Glasgow coma scale, mRS modified Rankin scale, MRI magnetic resonance imaging, NIHSS National Institutes of Health Stroke Scale, OR odds ratio.

*For every 10-unit increase.

†night time, weekend, and public holidays.

**Figure 1:**
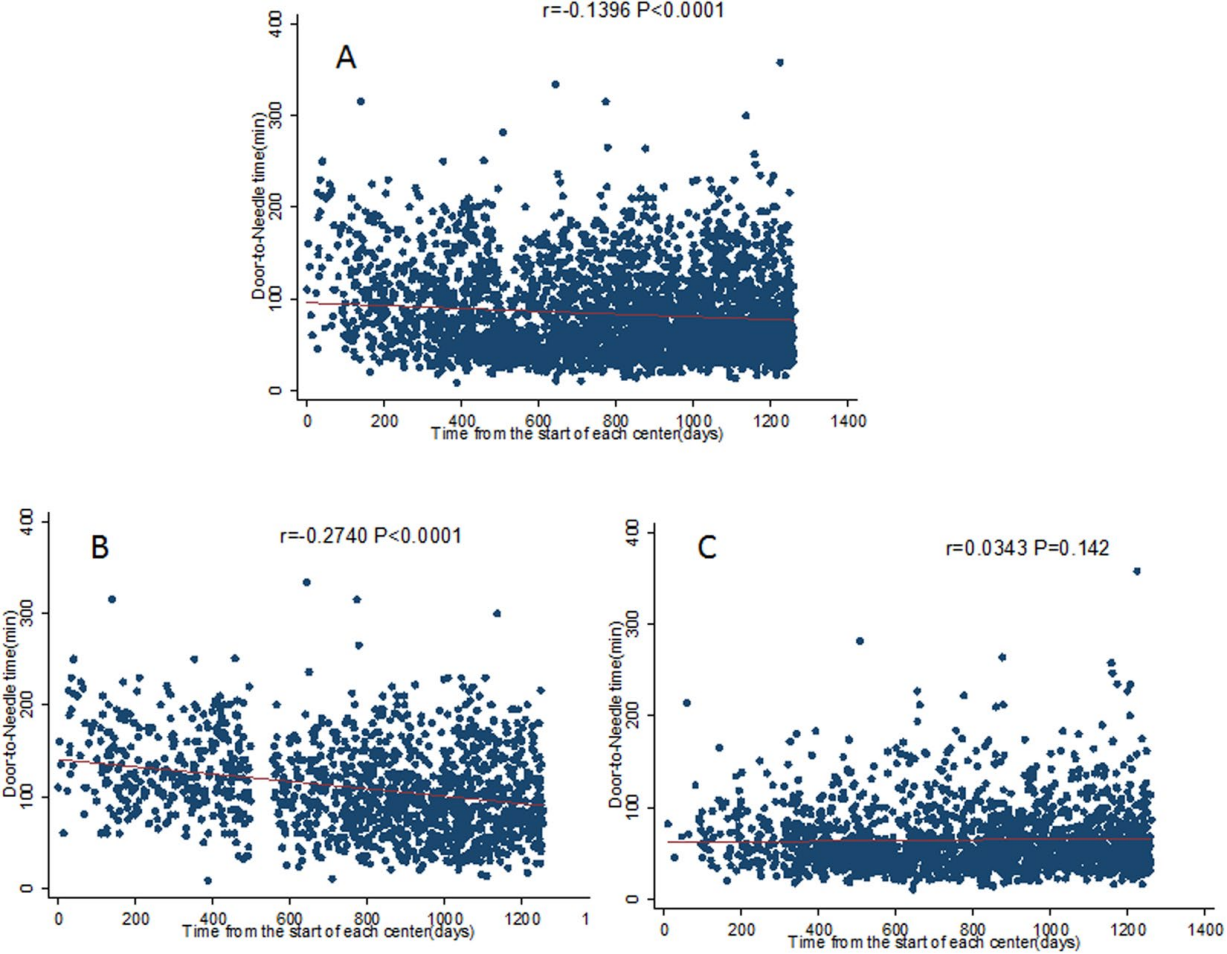
Correlation between time from the activation of each center in the ENCHANTED trial and door-to-needle time for (**A**) all patients, (**B**) Chinese patients, and (**C**) non-Chinese patients.

Table 2 and Fig. 1 show that the overall mean DNT (min) was 71.8 (95%CI 70.4–73.2), and that it decreased significantly over the course of the trial (fourths): 77.9 (74.9–80.9), 69.3 (66.7–72.0), 69.1 (66.5–71.8) and 71.4 (68.7–74.2) (P for trend, 0.003) and this trend remained consistent after adjusting for confounders. In particular, there was a significant reduction of 33 minutes between the first and final fourth epocs in time across the trial in China (P for trend, 0.001), whilst there was little temporal difference in the other participating countries.

**Table 2:** Door-to-Needle time over time.

Fourth of time from the start of each center (days)	Unadjusted analysis		P value for interaction	Adjusted analysis*		P value for interaction	Median (IQR)
	Mean (95%CI)	P value for interaction		Mean (95%CI)	P value for interaction		
**Overall**	71.8 (70.4–73.2)	0.003			0.003		72 (48–108)
0–534	77.9 (74.9–80.9)			78.2 (75.5–81.1)			78 (48–120)
535–839	69.3 (66.7–72.0)			70.3 (67.8–72.9)			72 (48–108)
840–1056	69.1 (66.5–71.8)			70.0 (67.5–72.6)			72 (48–102)
1057–1262	71.4 (68.7–74.2)			73.0 (70.4–75.7)			72 (48–108)
**China (n** = **1382)**	97.5 (95.0–100.1)	<0.001	<0.001		<0.0001	<0.001	102 (78–138)
0–534	123.0 (116.8–129.5)			115.1 (107.9–122.7)			126 (102–168)
535–839	98.7 (93.7–103.9)			101.6 (95.1–108.6)			108 (78–138)
840–1056	84.8 (80.8–89.0)			92.3 (86.5–98.5)			90 (60–120)
1057–1262	90.2 (85.9–94.8)			95.6 (89.5–102.1)			96 (66–132)
**Non-China (n** = **1837)**	57.1 (55.8–58.3)	0.065			0.083		54 (42–78)
0–534	56.8 (54.4–59.4)			56.5 (54.0–59.1)			54 (42–78)
535–839	54.6 (52.2–57.0)			52.7 (50.4–55.2)			54 (42–78)
840–1056	57.7 (55.1–60.4)			56.7 (54.1–59.5)			60 (42–78)
1057–1262	59.4 (56.8–62.1)			58.5 (55.8–61.3)			60 (42–78)

CI denotes confidence interval; IQR denotes InterQuartile Range.

*Adjusted for time from onset to hospital arrival, sex, baseline diastolic blood pressure, Off-hour admission (night time, weekend, and public holidays), No. of patients treated in the center, and CT or MRI angiogram showing proximal occlusion.

## Discussion

These analyses of a large clinical trial dataset involving AIS patients treated in a wide range of hospitals in multiple countries shows that DNT, whilst longer than recommended in best practice guidelines, progressively decreased in centers over the course of the study. In particular, centers in China achieved a 33 minutes reduction in DNT over three years of participation in ENCHANTED, whilst DNT remained consistent elsewhere. Aside from recruitment in China, the independent predictors of long DNT included shorter ODT, fewer patients treated at a center, higher diastolic BP, off-hour admission, and the absence of proximal occlusion on imaging.

Our analysis demonstrated that there has been a significant reduction in DNT over time for patients in China, which was not matched by reductions in other regions. However, there is still a large gap between the DNT in China (mean DNT 97.5 min, which is consistent with some previous observational studies in China) and the quality performance DNT of <60 min recommended in international guidelines^8–11^. Although various quality improvement efforts have been undertaken in China in the last decade, resulting in dramatic improvements in acute stroke care, there remains significant gaps between guideline recommendations and clinical practice in this country^9^. The Target Stroke Initiative, launched by the American Heart Association and American Stroke Association in 2011, was successful at improving the timeliness of rtPA administration for AIS on a national scale in the United States^10,12,13^. A similar comprehensive program is clearly needed in China, with ongoing monitoring of DNT as an important benchmark for quality in stroke care^1,10–12^. Why DNT did not improve for non-China centers (mean 57.1 min) may relate to the already strong performance and low volumes compared with Chinese centers.

Our analyses identified some interesting factors influencing DNT. Firstly, our data confirm the paradox that shorter ODT is associated with longer DNT^13–15^. This suggests that the responsible medical officer may be influenced by the perception that there is ample time prior to the expiration of the thrombolysis window - this may be especially true in China^12,16^. Secondly, in line with previous reports^14,17^, we found that hospitals with higher annual volumes of thrombolysis activity achieved significantly shorter DNT. This may relate to high-volume hospitals having better processes in place for investigation and decision-making for rtPA administration^17^. Thirdly, our analysis of higher diastolic BP being associated with longer DNT supports the findings of Skolarus *et al*. who reported that pre-thrombolytic antihypertensive treatment for patients with elevated BP prolongs DNT in a secondary analysis of a clinical trial population^18^. Furthermore, our analysis demonstrates that off-hour admission was an independent predictor of longer DNT, which may be due to reduced hospital staffing and delayed imaging procedures during off-hour time^19,20^.

Key strengths of our study include the large sample size of patients, and the rigorous evaluation^7,8^. However, as ENCHANTED excluded those patients who were likely to die within the next 24 hours, the present findings may not be generalizable to patients with severe AIS. In addition, several factors which influence DNT, including hospital pre-notification by ambulances^10^ and time needed for informed consent and randomization, were not collected and thus excluded from the adjusted analyses.

In summary, DNT progressively declined among centers participating in the ENCHANTED trial, especially in China. However, as DNT remains longer than the 60 minute cut-point recommended in guidelines, national programs to improve the timeliness of delivery of rtPA are needed in China.

## Methods

### Design

The ENCHANTED trial is an international, multi-center, prospective, randomized, open-label, blinded-endpoint trial; the details of which are outlined elsewhere^7,8,21^. In brief, 3310 patients with a clinical diagnosis of AIS confirmed on brain imaging and fulfilling local criteria for thrombolysis treatment, including symptom onset within 4.5 hours, participated in the rtPA dose evaluation arm, where they were randomly assigned to receive low- (0.6 mg/kg; 15% as bolus, 85% as infusion over 1 hour) or standard-dose (0.9 mg/kg; 10% as   bolus, 90% as infusion over 1 hour) rtPA. The study protocol was approved by Ethics Committee of Peking University First Hospital and appropriate ethics committee at each participating center. Written informed consent was obtained from each patient or their appropriate surrogate. Participants of this study were managed according to standard guidelines and protocols at each hospital.

### Procedures

Key demographic and clinical characteristics were recorded at the time of enrolment, with stroke severity measured using the National Institute of Health Stroke Scale (NIHSS) and Glasgow Coma Scale (GCS) at baseline, 24 hours, and at Day 7 (or earlier on discharge from hospital). Uncompressed digital images of all baseline and follow-up digital CT, MRI and angiogram images were collected in DICOM format on a CD-ROM identified only with the patient’s unique study number, and uploaded by a special purpose-built web-based system for central analysis at The George Institute of Global Health.

### Statistical Analysis

Independent predictors of long DNT (>60 min) were determined using logistic regression models. Significant predictors (P < 0.05) from the univariate analysis were tested for their association with long DNT (>60 min) in a multivariable model. We reduced the full model by successively removing nonsignificant covariates until all the remaining predictors remained statistically significant (P < 0.05). Correlation between time from the activation of each center and DNT was assessed using Spearman Correlation. DNT was log-transformed to remove skewness. Generalized linear model was used to investigate secular trends in DNT across fourths of time since the start of each site with adjustment for all independent predictor of long DNT.

### Data availability

The dataset analysed in the current study are available upon appropriate request sent to a corresponding author.

## Electronic supplementary material

supplementary information
