# Supplementary material for: Positive impact of the participation in the ENCHANTED trial in reducing Door-to-Needle Time
Source: Sci Rep. 2017 Oct 26;7:14168. doi: 10.1038/s41598-017-14164-8 (PMC5658430; doi:10.1038/s41598-017-14164-8)
Supplement: Supplementary file 1 — supplementary information [file 41598_2017_14164_MOESM1_ESM.doc]

**Positive impact of the participation in the ENCHANTED trial in reducing Door-to-Needle**

Jie Yang^1, 2^* MD PhD, Xia Wang^2^ PhD, Jian ping Yu^1^ MD, Jing Hang^3^ MD, Pablo Lavados^4^ MD, Thompson Robinson^5^ MD, Hisatomi Arima^6^ PhD, Richard I Lindley^2^ MD, Craig S Anderson^7^* MD PhD, John Chalmers^2^ MD PhD

1 Department of Neurology, the First Affiliated Hospital of Chengdu Medical College, Chengdu, China.

2 The George Institute for Global Health, Faculty of Medicine, University of New South Wales, Sydney, Australia.

3 Department of Neurology, Nanjing First Hospital, Nanjing Medical University, Nanjing, China.

4 Servicio de Neurología, Departamento de Medicina, ClínicaAlemana, Universidad del Desarrollo, and Universidad de Chile, Santiago, Chile.

5 Department of Cardiovascular Sciences and NIHR Biomedical Research Centre, University of Leicester, Leicester, UK.

6 Department of Public Health, Fukuoka University, Japan.

7 The George Institute for Global Health at Peking University Health Science Center, Beijing, China.

***Co-Authors for correspondence:**

Jie Yang MD, PhD

Department of Neurology

The First Affiliated Hospital of Chengdu Medical College.

No.278, BaoguangDadaoZhongduan Road, Xindu District, Chengdu 610599, China

Email:yangjie1126@163.com

Craig Anderson MD, PhD

The George Institute for Global Health at Peking University Health Science Center

PO Box Level 18, Tower B, Horizon Tower, No. 6 Zhichun Rd Haidian District, Beijing, 100088 P.R. China

Email: canderson@georgeinstitute.org.cn

**Table S1**. Baseline characteristics of Chinese patients according to door-to-needle time above and below 60 minutes

|  | DNT ≤60 mins (n=235) | DNT >60 mins  (n=1147) | *P* value | OR (95%CI) | aOR (95%CI) | *P* value |
| --- | --- | --- | --- | --- | --- | --- |
| Age, years | 66 (11) | 64 (12) | 0.091 |  |  |  |
| Female | 71 (30) | 401 (35) | 0.162 |  |  |  |
| Clinical features |  |  |  |  |  |  |
| Systolic BP, mmHg | 153 (18) | 150 (19) | 0.026 | 0.92 (0.85-0.99)* |  |  |
| Diastolic BP, mmHg | 86 (11) | 88 (13) | 0.023 |  |  |  |
| Heart rate, beats per minute | 77 (12) | 78 (14) | 0.200 |  |  |  |
| NIHSS score | 8 (4-12) | 9 (5-14) | 0.009 | 1.04 (1.02-1.06) |  |  |
| ≥14 | 46 (20) | 303 (26) | 0.028 |  |  |  |
| GCS score | 15 (14-15) | 15 (13-15) | 0.245 |  |  |  |
| Severe (3-8) | 9 (4) | 76 (7) | 0.104 |  |  |  |
| Medical history |  |  |  |  |  |  |
| Hypertension | 146 (62) | 696 (61) | 0.679 |  |  |  |
| Previous stroke | 40 (17) | 181 (16) | 0.636 |  |  |  |
| Coronary artery disease | 8 (3) | 55 (5) | 0.352 |  |  |  |
| Other heart disease | 32 (14) | 152 (13) | 0.881 |  |  |  |
| Atrial fibrillation | 35 (15) | 178 (16) | 0.805 |  |  |  |
| Diabetes mellitus | 50 (21) | 224 (20) | 0.541 |  |  |  |
| Hypercholesterolemia | 9 (4) | 48 (4) | 0.803 |  |  |  |
| Current smoker | 71 (30) | 292 (26) | 0.131 |  |  |  |
| Pre-stroke function (mRS) |  |  |  |  |  |  |
| No symptoms | 211 (90) | 1000 (87) | 0.283 |  |  |  |
| No significant disability | 24 (10) | 146 (13) |  |  |  |  |
| Medications |  |  |  |  |  |  |
| Antihypertensive agent(s) | 85 (36) | 418 (36) | 0.937 |  |  |  |
| Warfarin anticoagulation | 2 (1) | 10 (1) | 0.975 |  |  |  |
| Aspirin/other antiplatelet agent(s) | 27 (12) | 156 (14) | 0.384 |  |  |  |
| Glucose-lowering treatment | 28 (12) | 134 (12) | 0.920 |  |  |  |
| Statin/other lipid lowering | 13 (6) | 66 (6) | 0.894 |  |  |  |
| Brain imaging features |  |  |  |  |  |  |
| Visible early ischemic changes | 17 (7) | 153 (13) | 0.009 |  |  |  |
| Visible cerebral infarction | 20 (9) | 215 (19) | 0.0001 |  |  |  |
| Visible cerebral infarction with mass effect | 4 (2) | 27 (2) | 0.539 |  |  |  |
| CT/MRI angiogram shows proximal occlusion | 7 (3) | 51 (4) | 0.307 |  |  |  |
| Final diagnosis at time of hospital separation | N=231 | N=1127 |  |  |  |  |
| Non-stroke | 2 (1) | 6 (1) | 0.191 |  |  |  |
| Large artery occlusion due to significant atheroma | 114 (49) | 656 (58) |  |  |  |  |
| Small vessel or perforating vessel lacunar disease | 74 (32) | 279 (25) |  |  |  |  |
| Cardioembolism | 27 (12) | 117 (10) |  |  |  |  |
| Dissection | 1 (0) | 3 (0) |  |  |  |  |
| Other or uncertain etiology | 13 (6) | 66 (6) |  |  |  |  |
| Off-hour admission† | 151 (64) | 766 (67) | 0.455 |  |  |  |
| No. of patients treated in the center | 189 (71-279) | 126 (43-189) | <0.0001 | 0.94(0.92-0.95) | 0.95(0.93-0.96) | <0.001 |
| Time from onset to door, hour | 2.3 (1.5-3.0) | 1.2 (0.8-1.8) | <0.0001 | 0.29 (0.24-0.34) | 0.30 (0.25-0.37) | <0.001 |

Data are n (%), mean (SD), or median (IQR)· P values based on Chi-square, T test, or Wilcoxon signed-rank test

aOR denotes adjusted odds ratio, BP blood pressure, CI confidence interval, CT computerized tomography, GCS Glasgow coma scale, mRS modified Rankin scale, MRI magnetic resonance imaging, NIHSS National Institutes of Health Stroke Scale, OR odds ratio

*for every 10-unit increase

†night time, weekend, and public holidays

**Table S2**. Baseline characteristics of non-Chinese participants according to door-to-needle time above and below 60 minutes

|  | DNT≤ 60m (n=1081) | DNT>60m (n=756) | P value | OR (95%CI) | AOR (95%CI) | P value |
| --- | --- | --- | --- | --- | --- | --- |
| Age (years) | 68.1 (13.1) | 68.5 (13.9) | 0.571 |  |  |  |
| Female | 408/1081 (37.7) | 341/ 756 (45.1) | 0.002 | 1.36(1.12-1.64) | 1.34(1.11-1.63) | 0.003 |
| Region of recruitment |  |  |  |  |  |  |
| Asians, other than Chinese | 423/1081 (39.1) | 232/ 756 (30.7) | 0.0002 | 0.69(0.57-0.84) | 0.62(0.51-0.76) | <0.0001 |
| Others | 658/1081 (60.9) | 524/ 756 (69.3) |  | 1.0 |  |  |
| Clinical features |  |  |  |  |  |  |
| Systolic BP (mmHg) | 147.4 (20.8) | 149.1 (20.2) | 0.091 |  |  |  |
| Diastolic BP (mmHg) | 82.5 (12.9) | 82.8 (12.9) | 0.607 |  |  |  |
| Heart rate (beats per minute) | 80.2 (16.1) | 80.2 (17.1) | 0.904 |  |  |  |
| NIHSS score | 8.0 (5.0 - 13.0) | 8.0 (5.0 - 15.0) | 0.100 |  |  |  |
| ≥ 14 | 269/1081 (24.9) | 213/ 756 (28.2) | 0.115 |  |  |  |
| GCS score | 15.0 (14.0 - 15.0) | 15.0 (14.0 - 15.0) | 0.180 |  |  |  |
| Severe (3-8) | 24/1081 (2.2) | 23/ 756 (3.0) | 0.272 |  |  |  |
| Medical History |  |  |  |  |  |  |
| Hypertension | 676/1081 (62.5) | 498/ 756 (65.9) | 0.143 |  |  |  |
| Previous stroke | 137/1081 (12.7) | 111/ 756 (14.7) | 0.215 |  |  |  |
| Coronary artery disease | 95/1081 (8.8) | 75/ 756 (9.9) | 0.410 |  |  |  |
| Other heart disease (valvular or other) | 159/1081 (14.7) | 118/ 756 (15.6) | 0.596 |  |  |  |
| Atrial fibrillation | 236/1080 (21.9) | 175/ 755 (23.2) | 0.502 |  |  |  |
| Diabetes mellitus | 211/1081 (19.5) | 144/ 756 (19.0) | 0.801 |  |  |  |
| Hypercholesterolaemia | 282/1081 (26.1) | 207/ 756 (27.4) | 0.537 |  |  |  |
| Current smoker | 240/1079 (22.2) | 155/ 754 (20.6) | 0.388 |  |  |  |
| Pre-stroke function (mRS) |  |  |  |  |  |  |
| No symptoms | 848/1081 (78.4) | 561/ 755 (74.3) | 0.039 | 1.26(1.01-1.57) |  |  |
| No significant disability | 233/1081 (21.6) | 194/ 755 (25.7) |  | 1.0 |  |  |
| Medication at time of admission |  |  |  |  |  |  |
| Antihypertensive agents | 550/1081 (50.9) | 408/ 756 (54.0) | 0.192 |  |  |  |
| Warfarin anticoagulation | 30/1078 (2.8) | 36/ 756 (4.8) | 0.025 | 1.75(1.07-2.86) |  |  |
| Aspirin or other anti-platelet agents | 317/1078 (29.4) | 232/ 756 (30.7) | 0.555 |  |  |  |
| Glucose-lowering agents | 136/1078 (12.6) | 104/ 756 (13.8) | 0.476 |  |  |  |
| Statin or other lipid lowering agent | 324/1078 (30.1) | 201/ 755 (26.6) | 0.110 |  |  |  |
| Brain imaging features |  |  |  |  |  |  |
| Visible early ischemic changes | 352/1081 (32.6) | 237/ 756 (31.3) | 0.584 |  |  |  |
| Visible cerebral infarction | 321/1081 (29.7) | 172/ 756 (22.8) | 0.001 |  |  |  |
| Visible cerebral infarction with mass effect | 15/1081 (1.4) | 1/ 756 (0.1) | 0.004 |  |  |  |
| CT or MRI angiogram showing proximal occlusion | 288/1055 (27.3) | 152/ 741 (20.5) | 0.001 |  |  |  |
| Final diagnosis at time of hospital separation |  |  |  |  |  |  |
| Non-stroke | 49/1069 (4.6) | 28/ 750 (3.7) | 0.061 |  |  |  |
| Large artery occlusion due to significant atheroma | 307/1069 (28.7) | 177/ 750 (23.6) |  |  |  |  |
| Small vessel or perforating vessel lacunar disease | 187/1069 (17.5) | 125/ 750 (16.7) |  |  |  |  |
| Cardioembolism | 274/1069 (25.6) | 214/ 750 (28.5) |  |  |  |  |
| Dissection | 14/1069 (1.3) | 7/ 750 (0.9) |  |  |  |  |
| Other or uncertain aetiology | 238/1069 (22.3) | 199/ 750 (26.5) |  |  |  |  |
| Off-hour admission (night time, weekend, and public holidays) | 476/1081 (44.0) | 387/ 756 (51.2) | 0.003 | 1.33(1.11-1.61) | 1.44(1.19-1.75) | 0.0002 |
| No. of patients treated in the center | 51.0 (29.0 - 80.0) | 42.0 (16.0 - 88.0) | <.0001 | 0.98(0.96-1.00) |  |  |
| Time from onset to door | 1.4 (0.9 - 2.1) | 1.3 (0.8 - 1.9) | 0.001 | 0.79(0.71-0.89) | 0.77(0.68-0.86) | <0.0001 |

Data are n (%), mean (SD), or median (IQR)· The P values are based on Chi-square, T test, or Wilcoxon signed-rank test

AOR: adjusted odds ratio; BP: blood pressure; CI: confidence interval; CT: computed tomography; GCS: Glasgow Coma Scale; mRS: modified Rankin scale; MRI: Magnetic resonance imaging; NIHSS: The National Institutes of Health Stroke Scale; OR: odds ratio

*for every 10-unit increase
